# Supplementary material for: Euprymna berryi as a comparative model host for Vibrio fischeri light organ symbiosis
Source: Appl Environ Microbiol. 2025 Jul 10;91(8):e00001-25. doi: 10.1128/aem.00001-25 (PMC12366355; doi:10.1128/aem.00001-25)
Supplement: Supplemental material — Table S1 and Figures S1 to S5. [file aem.00001-25-s0001.pdf]

Euprymna berryi as a comparative model host for _Vibrio fischeri_ light organ symbiosis

Avery M. Imes[1,2], Morgan N. Pavelsky[3], Klodia Badal[3], Derrick L. Kamp[4], John L. Briseño[4], Taylor Sakmar[5], Miranda A. Vogt[5], Spencer V. Nyholm[4], Elizabeth A. C. Heath-Heckman[6], Bret Grasse[5], Alecia N. Septer[3], Mark J. Mandel[1,2]

[1] Department of Medical Microbiology and Immunology, University of Wisconsin-Madison, Madison, WI USA

[2] Genetics Training Program, University of Wisconsin-Madison, Madison, WI USA

[3] Department of Earth, Marine & Environmental Sciences, University of North Carolina, Chapel Hill, NC USA

[4] Department of Molecular and Cell Biology, University of Connecticut, Storrs, CT USA

[5] Eugene Bell Center, Marine Biological Laboratory, Woods Hole, MA USA

[6] Department of Integrative Biology, Michigan State University, East Lansing, MI USA

SUPPLEMENTAL MATERIAL

Supplemental Table S1: Reference genomes used in FastANI analysis for species identification.

Species	Accession number
Pseudoalteromonas flavipulchra	ASM25911v1
Pseudoalteromonas maricaloris	ASM2474685v1
Vibrio atypicus (DSM 25292)	ASM981131v1
Vibrio campbellii	ASM290647v1
Vibrio fischeri (ES114)	ASM1180v1
Vibrio fischeri (MB14A3)	ASM164037v1
Vibrio fischeri (MJ11)	ASM2084v1
Vibrio harveyi	ASM3006043v1
Vibrio parahaemolyticus	ASM19609v1
Vibrio vulnificus	ASM222426v1

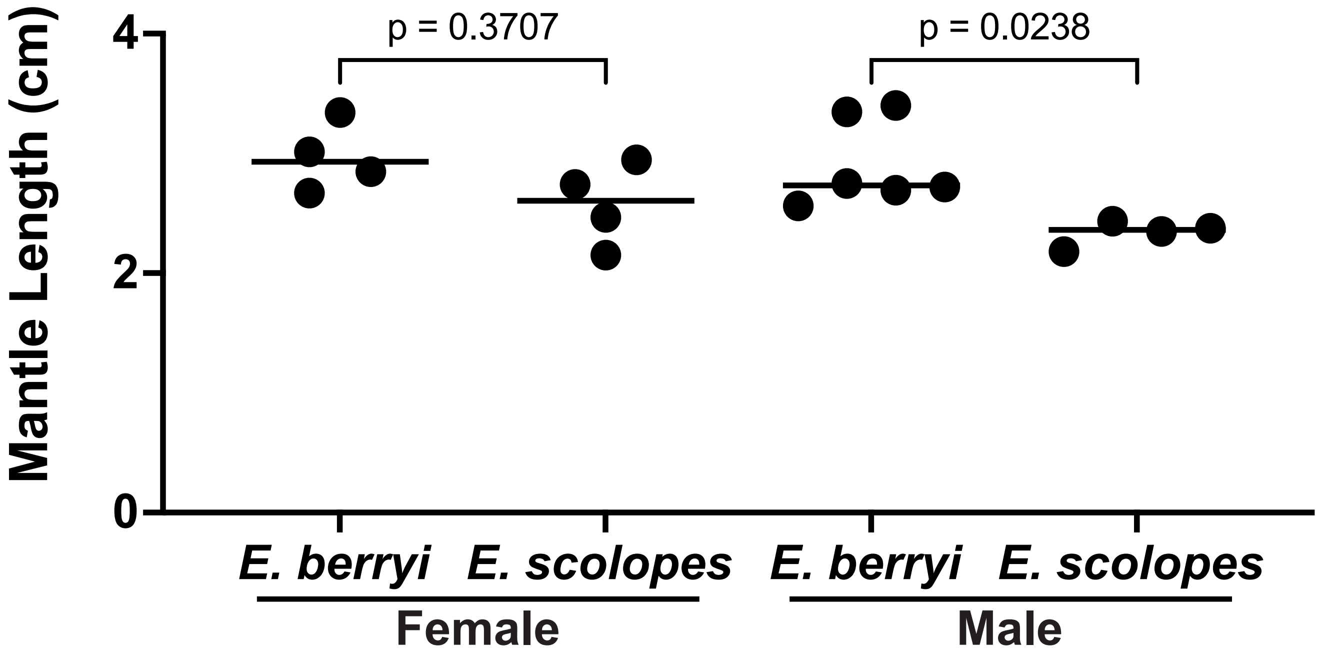

Supplemental Figure S1. Comparison of adult mantle length. Mantle lengths of adult *Euprymna* held in laboratory aquaculture. Mantle lengths were estimated in FIJI. Circles represent individual animals and the median is plotted (*n* = 4-6). A Kruskal-Wallis test was applied for statistical analysis. Among our small sample, we observed a statistically significant difference among males a trend among females where *E. berryi* was larger.

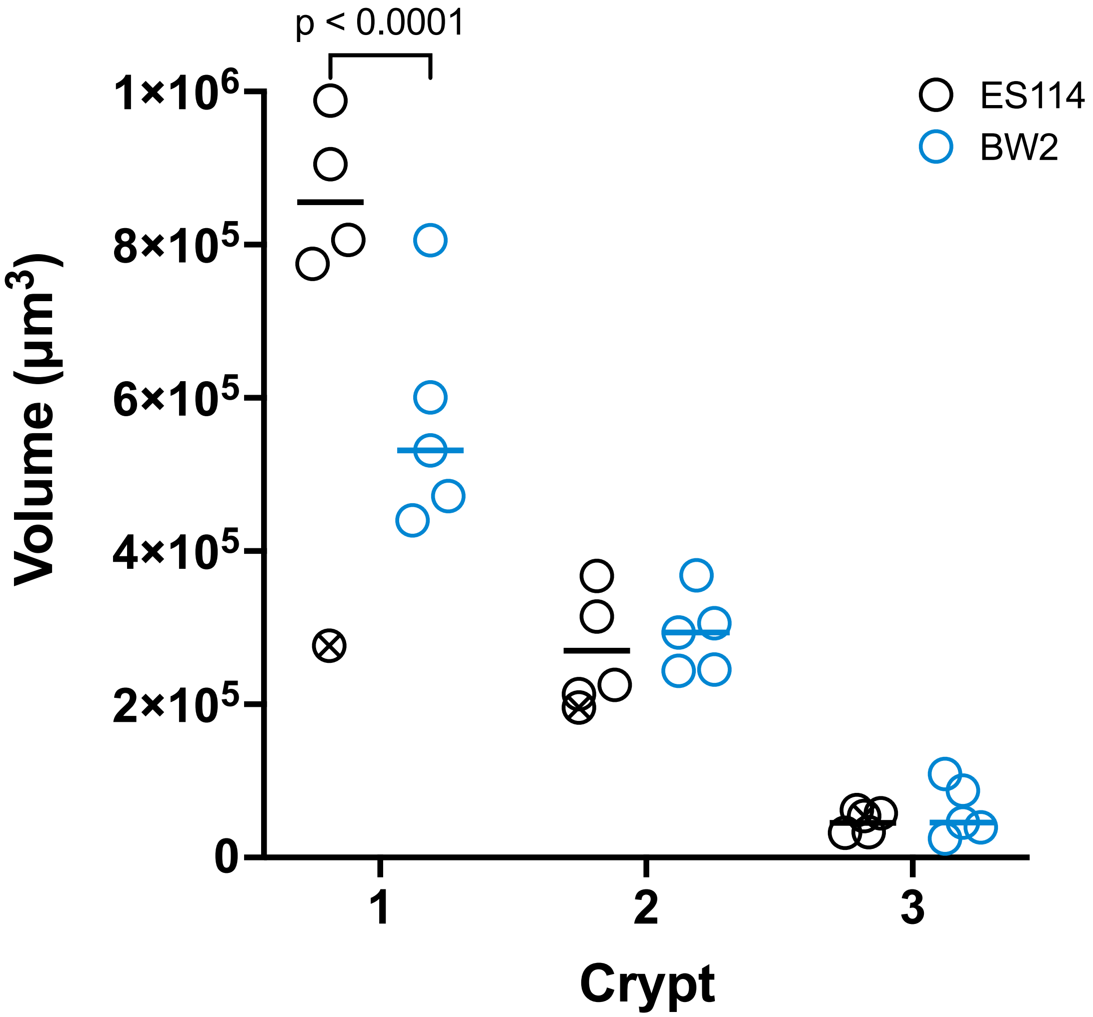

Supplemental Figure S2. ES114 occupies a greater crypt volume in crypt 1 than BW2. Z-stack images of single light organ lobes of *E. berryi* approximately 150 μm deep were taken at 1.2 μm intervals. Animals were colonized with a pVSV102 GFP-expressing derivative of the indicated strain. ES114 is a *V. fischeri* isolate from *E. scolopes,* and BW2 is a *V. fischeri* isolate from *E. berryi*. Image stacks were processed and analyzed using Arivis software, using the GFP signal to determine the space occupied by the *V. fischeri* and calculate the size of the crypt. The median of each group is shown with a horizontal bar (*n* = 4-5). ES8, an animal that vented its crypt bacteria during processing, is marked with an X and omitted from statistical analysis. Statistical analysis was performed using a two-way ANOVA test. Crypts 2 and 3 were not significantly different ($p > 0.99$).

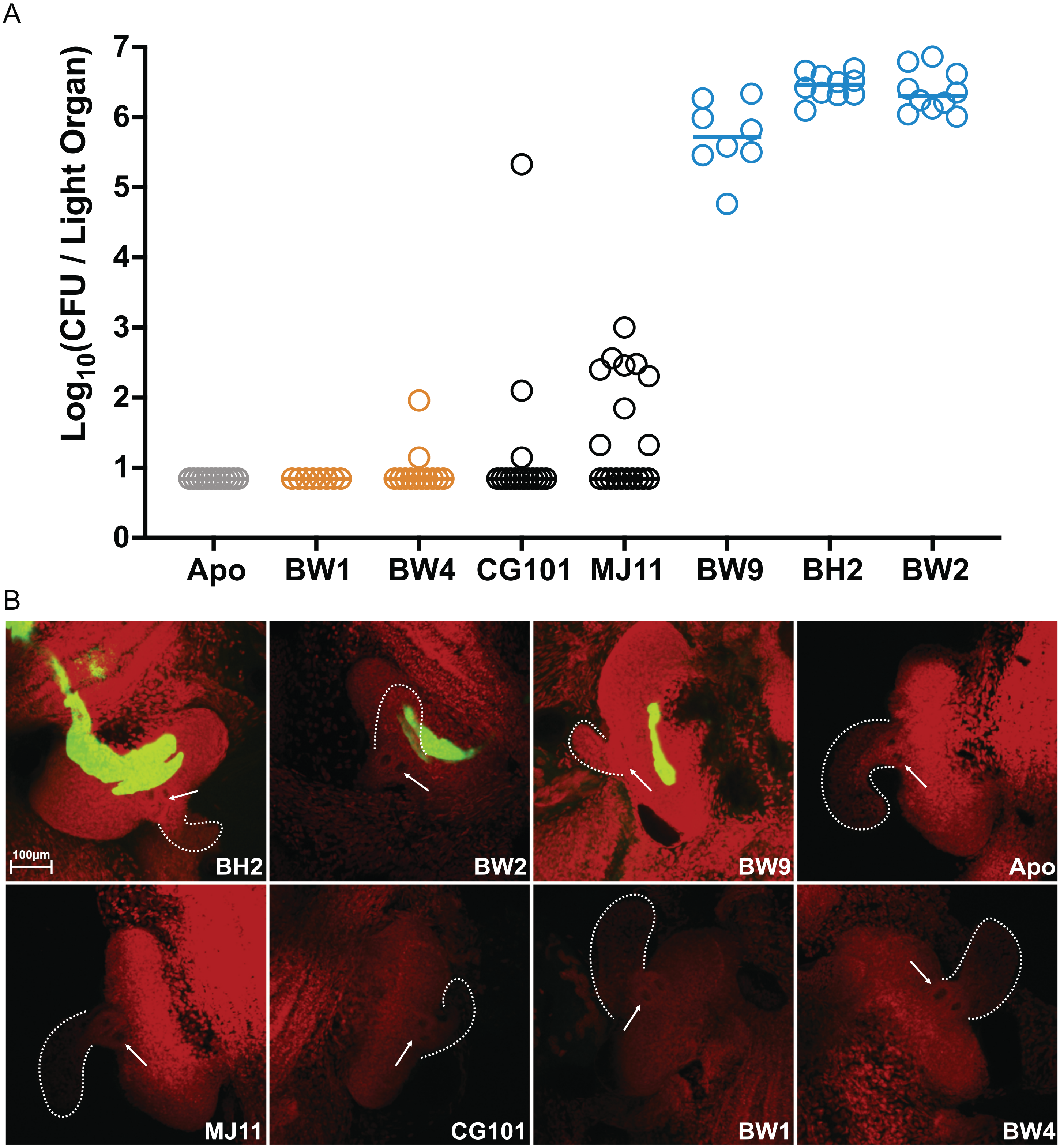

Supplemental Figure S3. Non-*V. fischeri* are not observed in the *E. berryi* light organ or surrounding tissues. (A) *E. berryi* hatchlings were used in single-strain colonization experiments, where circles represent individual animals. Colonization was estimated by dissection of the light organ and minimal surrounding tissue for homogenization and plating. The limit of detection for this assay, represented by the dashed line, is 7 CFU/light organ. Horizontal bars represent the median for each group (*n* = 12-19). (B) Representative images of *E. berryi* inoculated with the indicated GFP-expressing (green) bacterial strain (*n* = 4-10). Images are a maximum intensity projection of a confocal z-stack approximately 70 µm deep. Host tissue is stained with TO-PRO-3 (red). The anterior appendage is outlined in a dotted white line. The three pores of each lobe are indicated by a white arrow. All images have the same scale.

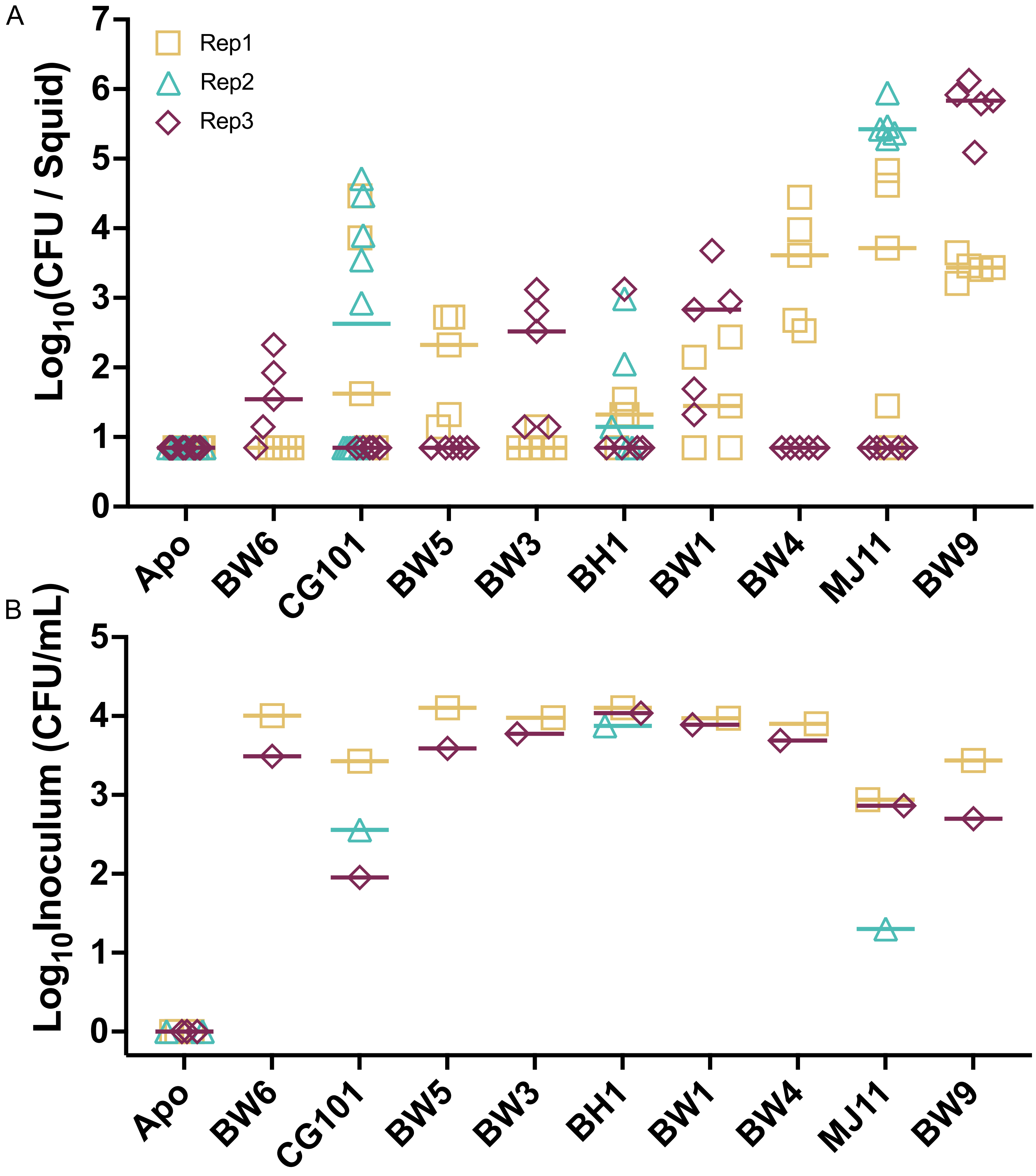

Supplemental Figure S4. Inoculum concentration did not positively correlate to squid colonization concentration in strains that exhibited bimodality. (A) A reproduction of monocolonization data for select strains as presented in Fig4. Each symbol represents an individual animal, where color and shape indicate the replicate group that animal belonged to. Apo represents aposymbiotic squid, which are not provided bacterial inoculum in these experiments. (B) The inoculum concentration provided to squid, where each symbol represents the concentration of the indicated strain provided to the squid group within an experiment. Horizontal bars represent the median in both graphs. Colors in panel A and B correspond to the same replicate across experiments.

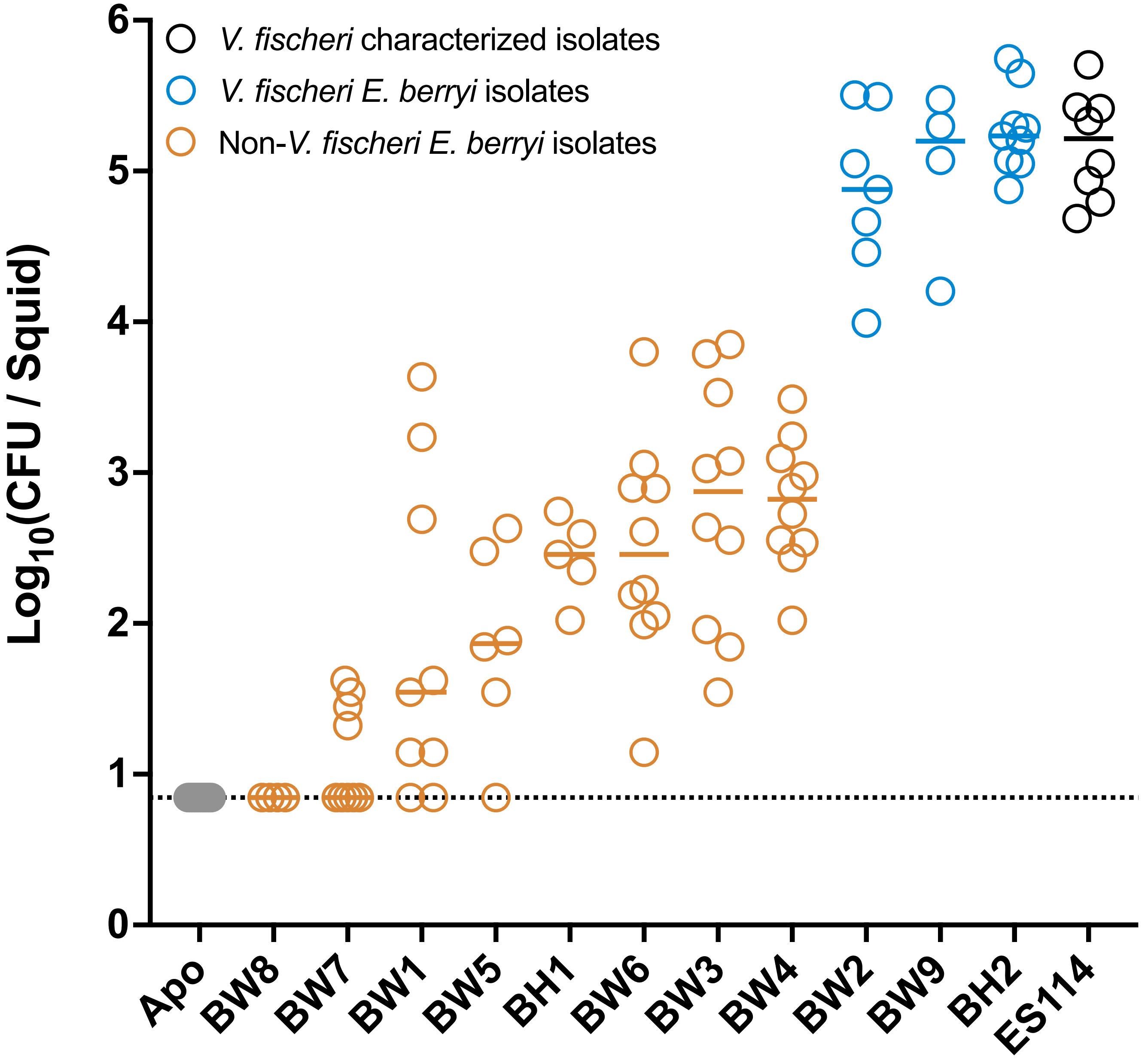

Supplemental Figure S5. *E. scolopes* are colonized by *V. fischeri* isolated from *E. berryi*. *E. scolopes* hatchlings were used in single-strain colonization experiments, where circles represent individual animals. The limit of detection for this assay, represented by the dashed line, is 7 CFU/light organ. Horizontal bars represent the median for each group (n = 4-13).
